# Iatrogenic caecal perforation two days after a caesarean section, a case report

**DOI:** 10.1016/j.amsu.2021.102924

**Published:** 2021-10-14

**Authors:** Donald Schweitzer, Anne-Claire Musters, Bart de Vries, Guy H.E.J. Vijgen

**Affiliations:** a Department of Surgery, Zuyderland Medisch Centrum, Heerlen, the Netherlands; b Department of Surgery, Maastricht University Medical Centre, Maastricht, the Netherlands; c Department of Gynecology, Zuyderland Medisch Centrum, Heerlen, the Netherlands; d Department of Pathology, Zuyderland Medisch Centrum, Heerlen, the Netherlands

**Keywords:** Caecal perforation, Pneumatosis intestinalis, Ileocolic anastomosis, Laparotomy

## Abstract

**Introduction:**

and importance: The caesarean section is a widely spread procedure and 29.7 million times performed every year inn 169 countries in the world. Overall, complications are seen in 6% for elective caesarean to 15% for emergency caesarean.

**Case presentation:**

We here report a case which was initially diagnosed as a postoperative paralytic ileus. After a complicated caesarean section caused by bleeding and problems with haemostasis, a healthy child was born with full mother recovery for the first 24 hours after surgery. Unfortunately, her condition deteriorated between 24 and 48 hours and she reported progressive nausea and painful bloating. Laboratory tests and CT imaging showed progressive signs of inflammation and distention of the caecum and colon. A second CT scan the next day revealed signs of perforation. An ileocecal resection was performed with a primary anastomosis. Full recovery occurred two weeks later.

**Clinical discussion:**

With an estimated incidence of only 0,08%, bowel perforations due to caesarean section, are rare. Moreover, is the clinical presentation diverse and computed topography is essential during the diagnostic process. To avoid potential morbidity and mortality, the surgeon must consider performing a laparotomy in case of a deteriorating patient in non-invasive treatment fails.

**Conclusion:**

Caecal perforation must be considered as complication after a caesarean section. An ileocecal resection is necessary in this situation. This case report shows that a primary anastomosis is a possible option in a healthy patient that is hemodynamically stable during the operation. In case of an unhealthy or hemodynamic unstable patient, the safest option is a temporary ileostomy.

## Highlights

1. Caecal perforation is a possible complication of a complicated caesarean section and should be part of the differential diagnosis in clinical detoriation.
2. If the condition of the women deteriorates do not hesitate to repeat CT or to perform an immediate surgical inspection.
3. A primary ileo-colic anastomosis could be a safe option in an otherwise healthy patient that is hemodynamically stable during surgery.

## 1 Introduction

The worldwide introduction of the caesarean section has been successful in reducing maternal and neonatal mortality. In general, it is a safe procedure with a low risk of iatrogenic damage. Iatrogenic gastro-intestinal injuries during a caesarean section are rare 0,08% [1]. Still, several case series have been reported on pseudo intestinal obstruction (Ogilvie's syndrome), which occurs 24–48 hours after surgery and can end in caecal or colonic blowout. We here report an intestinal perforation is an additional differential diagnosis. This case report has been reported according to the SCARE checklist in 2020 [2].

## 2 Case presentation

**Fig. 1:**
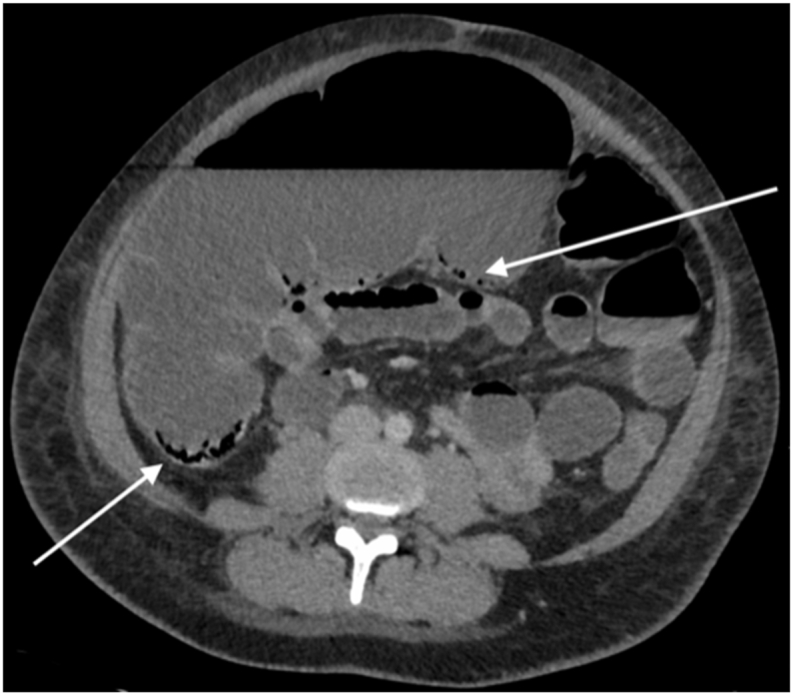
Transversal CT image: Distention of the colon of 10 cm, intestinal pneumatosis is indicated by 2 arrows.

A 32-year-old otherwise healthy non-smoking and non-drug using Caucasian nulliparous woman (BMI 22.2) with obstructed labour, underwent an emergency caesarean section. The procedure took 124 minutes with 2.0 L blood loss caused by insufficient uterine contraction and difficulties to obtain appropriate haemostasis. During the procedure two packed cells and one unit of fresh frozen plasma were infused. Moreover, 500 μg Sulprostone and 0,2 mg Methylergometrine was administered intravenously to promote contraction of the uterine arteries. In addition, Tranexamic acid 2 g. intravenously was administered to promote haemostasis. A healthy girl was born and eventually the mother was fine. Regrettably, her condition gradually deteriorated and at 48-h post-surgery the patient was seen by the consultant gastro-intestinal surgeon for a suspicion of paralytic ileus. Prior to this consultation, the abdomen was remarkably bloated but with no signs of peritonitis. Laboratory (Lab.) results disclosed *C*-Reactive Protein: 290 mg/L; Haemoglobin: 4.5 mmol/L and Leucocytosis: 28.7 × 10 9/L. Computer Tomography (CT) showed distended colon (max. 88 mm), (Fig. 1). A nasogastric tube was inserted and produced 500ml clear fluid during the following 12 hours.

### 2.1 Investigations

**Fig. 2:**
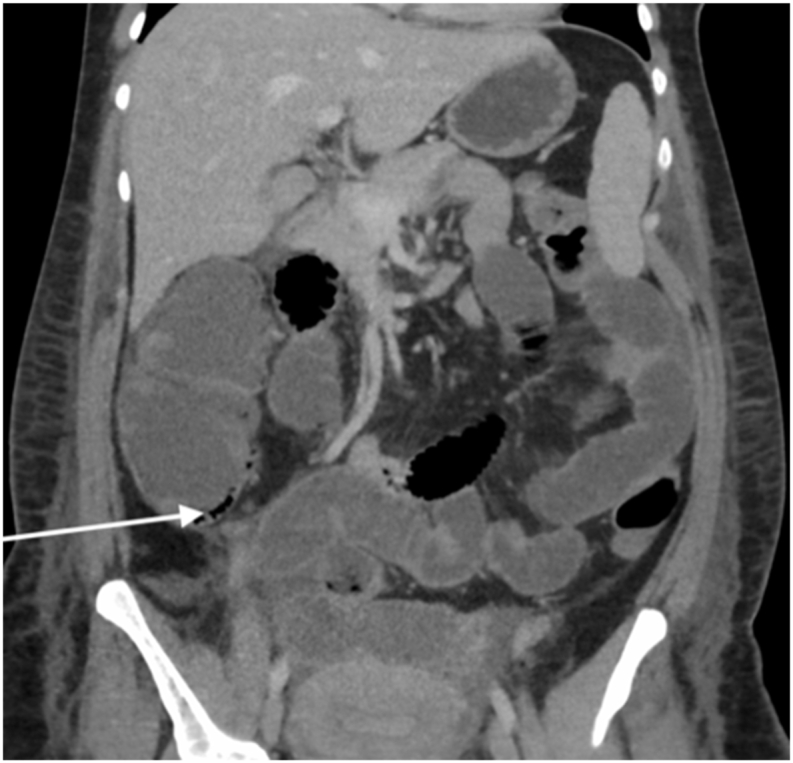
Coronal CT image, the largest diameter of the caecum is 10 cm, intestinal pneumatosis is indicated by 1 arrow.

Follow-up Laboratory tests the day after showed a further increase in inflammatory makers (*C*-Reactive Protein: 330 mg/L leukocytes: 38 × 10 9/L). A follow-up CT scan revealed progressive expansion of the caecum to 100 mm and was suggestive for intestinal pneumatosis without coexisting intraperitoneal gas, see Fig. 1, Fig. 2.

### 2.2 Differential diagnosis

The initial differential diagnosis was either transient paralytic ileus after caesarean section or progressive caecal and colonic distention based on pseudo-intestinal obstruction (Ogilvie's syndrome) [[3], [4], [5], [6], [7], [8], [9]]. However, a secondary CT scan the next day showed progression of distention and pneumatosis intestinalis [10,11]. By then, the differential diagnosis was either caecal blow-out or iatrogenic perforation that prompted surgical exploration.

### 2.3 Treatment

**Fig. 3:**
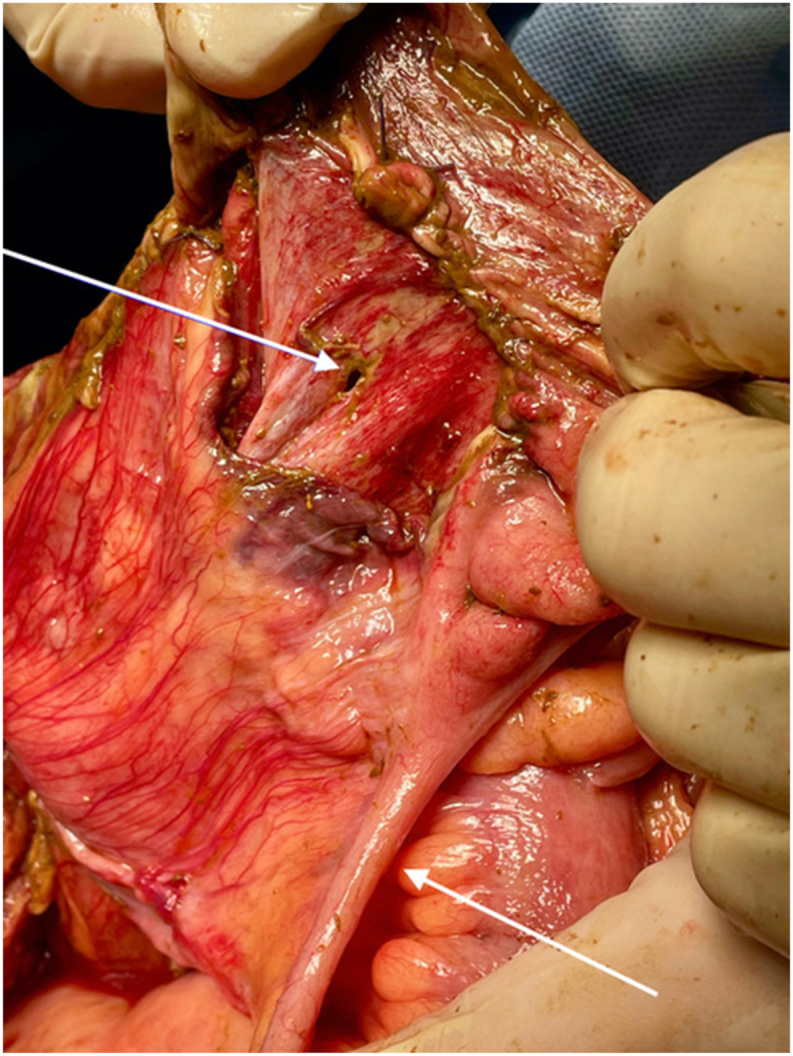
Per-operative photo, the healthy appendix and the perforation are indicated by an arrow.

A median laparotomy was performed 72 hours after the initial caesarean section. On inspection, there was a four-quadrant faecal peritonitis. The situs was flushed, and a careful inspection of the small and large intestine was performed. The coecum showed three linear full thickness perforations, possibly iatrogenic, see Fig. 3. An ileocecal resection was performed, followed by thorough rinsing with saline. At that moment, the quality and perfusion of the remaining small bowel and colon was good. We considered performing an ileostomy versus an anastomosis with or without a diverting ileostomy. Because of the hemodynamically stable situation and otherwise healthy patient we choose a primary side-to-side isoperistaltic ileo-colic anastomosis without a diverting ileostomy. No drains were left behind. The fascia was closed with PDS 2–0 and the skin with intracutaneous sutures. Ceftriaxone, Metronidazole, and Gentamycin were administered peri and post-surgery and continued for 2 weeks. Her condition gradually improved until full recovery.

## 3 Outcome and follow-up

Recovery lasted in total 14 days, 2 days at the ICU and 12 days at the surgical ward. A CT-scan performed 7-days post-surgery revealed free fluid in the abdominal cavity (right paracolic gutter), which needed ultrasound-guided drainage which revealed infected ascites. From then she received enteral nutrition, which she tolerated well.

**Fig. 4:**
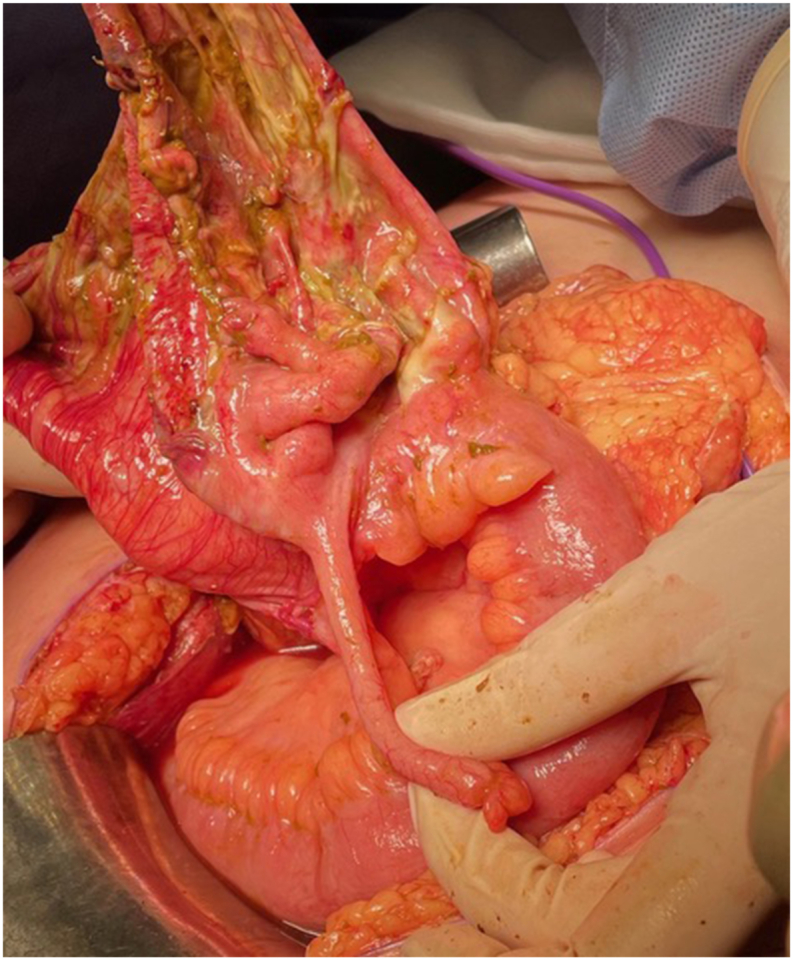
Per-operative photo, the healthy appendix and ileum.

At macroscopy, the resected ileocecal preparation showed a perforation in an expanded caecum (see Fig. 4). Histologically, there were signs of fibrinopurulent serositis suggestive of peritonitis. No signs of blowout, volvulus, ischemia, vasculitis, or thromboembolism. Macroscopic examination by the pathologist confirmed a perforation in a distended caecum, possibly caused by an accidental perforation of the caecum during the caesarean.

## 4 Discussion

Caesarean section is a very common obstetric procedure. The 2015 estimate of procedures worldwide is based on data from 169 countries is 29.7 million (21.1%, 95% Confidence Interval (CI):19.9–22.4) [12]. Despite this number, few updates have been published reporting complications other than maternal and/or neonatal mortality [13]. Post-caesarean paralytic ileus and pseudo intestinal obstruction (Ogilvie's syndrome) with or without colon perforation has been ascribed in particular to dilation of the bowel immediately after pregnancy due to rapidly decreasing concentrations of oestrogens and the anaesthetics administered during surgery and afterwards for pain relief [5,8,14,15]. To our knowledge, there have been no reports on peri-procedural perforations.

Caesarean sections can be complicated in case of massive bleeding. Therefore, detailed information about the course of the procedure is critical for careful post-caesarean patient management. In this case, it lasted 124 min. to complete the procedure, with a blood loss of more than 2.0 L. In contrast to paralytic ileus, pseudo intestinal obstruction with or without perforation (recognizable by an elongated tear of the intestine) has been reported in a case series of 21 women, 19 of them with tear lesion caused by mechanical force of distention of caecum or colon [5]. Another case report demonstrated a blowout of the caecum at day 4 after the caesarean section [8]. In this case, we decided to perform a primary ileocolic anastomosis based on the otherwise healthy bowel in a healthy young patient (Fig. 4). This worked out well.

In summary, iatrogenic intestinal perforation during a caesarean section may happen during a complicated procedure. This unfortunate event is currently not reported and therefore we here present this case that may be helpful in future clinical decision making.

## 5 Conclusion

To our knowledge, there are no other reported cases of caecal perforation during a caesarean section. In this healthy patient it was possible to perform an ileocolic anastomosis, the decision was based on the general condition and the hemodynamic stability during the operation. This case report is noteworthy due to the unusual complication, and successful surgical outcome that is not commonly seen.

### Patients’ perspective

I was under the influence of all medicines, I was afraid of the hospital and of undergoing (surgical) treatments. In addition, I found it very intense what happened in such a short time. My relationship with my baby is good. In addition, I have lost a bit of what exactly happened during the days before and after the surgical treatment. Now things are going well physically. But emotionally I still have to make steps, I did not expect that I would be left with a large scar. It will be fine, but it still takes time.

## Provenance and peer review

Not commissioned, externally peer reviewed.

## Consent for publication

Written informed consent was obtained from the patient for publication of this case report and accompanying images. A copy of the written consent is available for review by the Editor-in-Chief of this journal on request.

## Registration of research studies

Name of the registry:
Unique Identifying number or registration ID:
Hyperlink to your specific registration (must be publicly accessible and will be checked):

## Ethical approval

Not required.

## Funding

None.

## Research registration number

Not applicable.

## CRediT author contribution statement

Donald Schweitzer, Guy Vijgen = Study concept, Data collection, and surgical therapy for the patient.

Donald Schweitzer, Guy Vijgen, Anne-Claire Musters, Bart de Vries = Writing - original draft preparation.

Donald Schweitzer, Guy Vijgen, Anne-Claire Musters, Bart de Vries = Editing and writing.

Donald Schweitzer, Guy Vijgen = Senior author and manuscript reviewer.

All the authors read and approved the final manuscript.

## Declaration of competing interest

None.

## Guarantor

Donald Schweitzer accepts full responsibility for the work and/or the conduct of the study, has access to the data, and controls the decision to publish.

## Declaration of competing interest

None.
